# TLR2 and TLR4 Mediate Differential Responses to Limb Ischemia through MyD88-Dependent and Independent Pathways

**DOI:** 10.1371/journal.pone.0050654

**Published:** 2012-11-29

**Authors:** Ulka Sachdev, Xiangdong Cui, Ryan McEnaney, Tian Wang, Kelly Benabou, Edith Tzeng

**Affiliations:** 1 Surgical Service, Department of Veterans Affairs Medical Center, Pittsburgh, Pennsylvania, United States of America; 2 Department of Surgery, University of Pittsburgh Medical Center, Pittsburgh, Pennsylvania, United States of America; University of Otago, New Zealand

## Abstract

**Introduction:**

The danger signal HMGB1 is released from ischemic myocytes, and mediates angiogenesis in the setting of hindlimb ischemia. HMGB1 is a ligand for innate immune receptors TLR2 and TLR4. While both TLR2 and TLR4 signal through myeloid differentiation factor 88 (MyD88), TLR4 also uniquely signals through TIR-domain-containing adapter-inducing interferon-β (TRIF). We hypothesize that TLR2 and TLR4 mediate ischemic myocyte regeneration and angiogenesis in a manner that is dependent on MyD88 signaling.

**Methods:**

Mice deficient in TLR2, TLR4, MyD88 and TRIF underwent femoral artery ligation in the right hindlimb. Laser Doppler perfusion imaging was used to assess the initial degree of ischemia and the extent of perfusion recovery. Muscle regeneration, necrosis and fat replacement at 2 weeks post-ligation were assessed histologically and vascular density was quantified by immunostaining. In vitro, endothelial tube formation was evaluated in matrigel in the setting of TLR2 and TLR4 antagonism.

**Results:**

While control and TLR4 KO mice demonstrated prominent muscle regeneration, both TLR2 KO and TRIF KO mice exhibited marked necrosis with significant inflammatory cell infiltrate. However, MyD88 KO mice had a minimal response to the ischemic insult with little evidence of injury. This observation could not be explained by differences in perfusion recovery which was similar at two weeks in all the strains of mice. TLR2 KO mice demonstrated abnormal vessel morphology compared to other strains and impaired tube formation in vitro.

**Discussion:**

TLR2 and TRIF signaling are necessary for muscle regeneration after ischemia while MyD88 may instead mediate muscle injury. The absence of TLR4 did not affect muscle responses to ischemia. TLR4 may mediate inflammatory responses through MyD88 that are exaggerated in the absence of TLR2. Additionally, the actions of TLR4 through TRIF may promote regenerative responses that are required for recovery from muscle ischemia.

## Introduction

Peripheral artery disease (PAD) causes significant functional disability, leading to limb loss within six months of diagnosis in 25–40% of patients with unreconstructable disease. [1] Some patients with PAD develop adequate collateralization through angiogenesis that prevents the progression to tissue loss. [2], [3] The signals that promote these angiogenic processes are complex and not fully deciphered. While techniques to promote vessel growth by direct application of angiogenic factors into ischemic tissue have been studied and tested, [3], [4], [5], [6], [7], [8], [9], [10], [11] the results have been less than convincing, marked by the development of inadequate or immature vascular networks that are leaky. [9], [12] Studies using bone marrow or peripheral stem cells for limb salvage are ongoing and the efficacy of such cell based therapies has not yet been established. [13] Thus, further characterization of the signals that stimulate angiogenesis after ischemic injury and support maturation of these neovessels is necessary. Understanding the driving forces behind ischemia-induced angiogenesis will also contribute to the on-going development of endogenous revascularization strategies, potentially improving limb-salvage rates.

**Figure 1:**
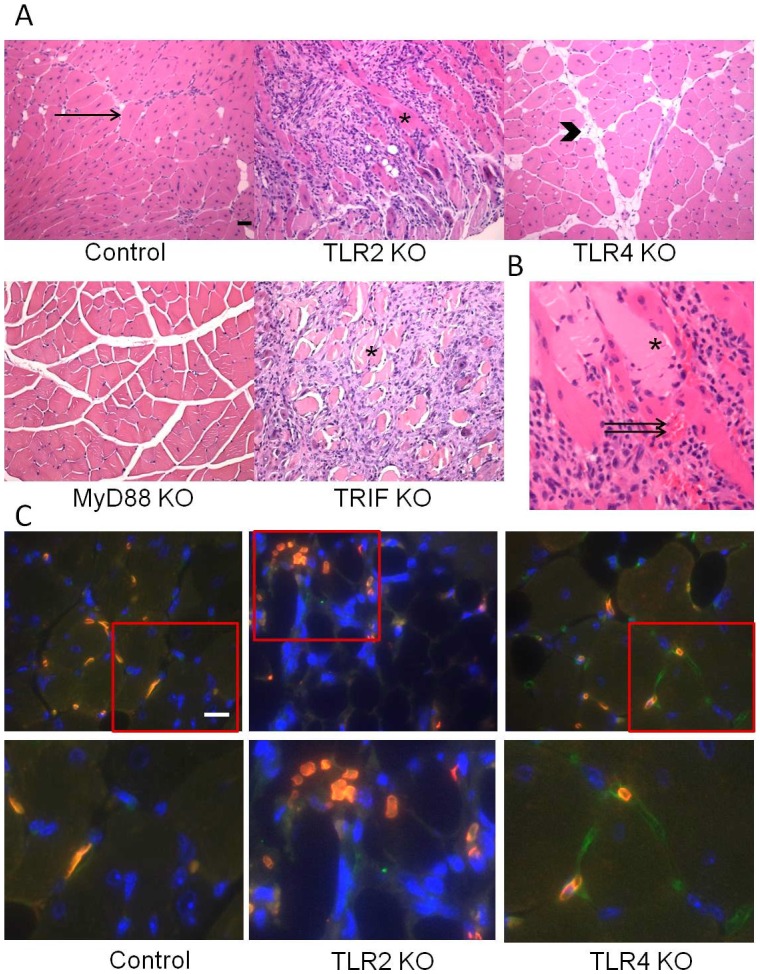
Skeletal muscle responses to hind limb ischemia at 2 weeks following femoral artery ligation. (A) Representative sections of tibialis anterior muscle from control C57B6, TLR2 KO, TLR4 KO, MyD88 KO and TRIF KO mice were stained with H&E. Arrow represents regenerating muscle fiber with centrally located nucleus. Arrowhead depicts areas of fat replacement. Asterisk indicates necrotic muscle fiber, characterized by eosinophilia, loss of muscle architecture and inflammatory infiltrate. (B) Hemorrhage into ischemic tissue was seen in TLR2KO mice (double arrow). Scale bar = 50 µm. (C) Erythrocytes (orange) are seen outside of vasculature (green) in TLR2KO mice. Red box demonstrates area of magnification shown in images below originals. Scale bar = 25 µm.

The innate immune system, characterized by highly conserved pattern-recognition receptors such as the toll-like receptors (TLRs) that respond to invading pathogens as well as endogenous injury ligands, has been shown in early studies to play an important role in angiogenesis. High Mobility Group Box-1 (HMGB1), a DNA binding protein that possesses cytokine like properties and can mediate systemic inflammatory processes through TLR activation, is released by necrotic cells and secreted by activated macrophages [14], . We have recently reported that HMGB1 is released by ischemic endothelial and skeletal muscle cells, mediates angiogenic behavior in endothelial cells (ECs), and increases limb perfusion in ischemic hind limbs. [17] The receptors for HMGB1 include TLR2, TLR4, and the Receptor for Advanced Glycation End-products (RAGE). While both TLR2 and TLR4 signal through myeloid differentiation factor 88 (MyD88), TLR4 also signals through TRIF (TIR-domain-containing adapter-inducing interferon-β). Given the potent effects of HMGB1 on EC angiogenic behavior in vitro and on ischemic hind limb perfusion recovery, we hypothesize that its receptors TLR2 and TLR4 are important in mediating the skeletal muscle and angiogenic responses to ischemic injury.

**Table 1:** Necrosis, fat replacement and muscle regeneration in control, TLR2 KO, and TLR4 KO mice two weeks following femoral artery ligation.

	Necrosis (%)	Fat (%)	Regenerating Muscle (%)
Control (N = 8)	0	7.4±1.3	93.4±1.2
TLR2 KO (N = 7)	19.2±7.8*	10.8±3.9	56.6±16.5**
TLR4 KO (N = 4)	0	11.9±2.18	86.0±1.2

Quantification of area was performed on 4–5 non-overlapping images per section with 3 sections per animal. Results are presented as % of total muscle area (mean ± SEM; *P<0.03 vs. control and TLR4 KO, **P<0.05 vs. control and TLR4 KO).

**Figure 2:**
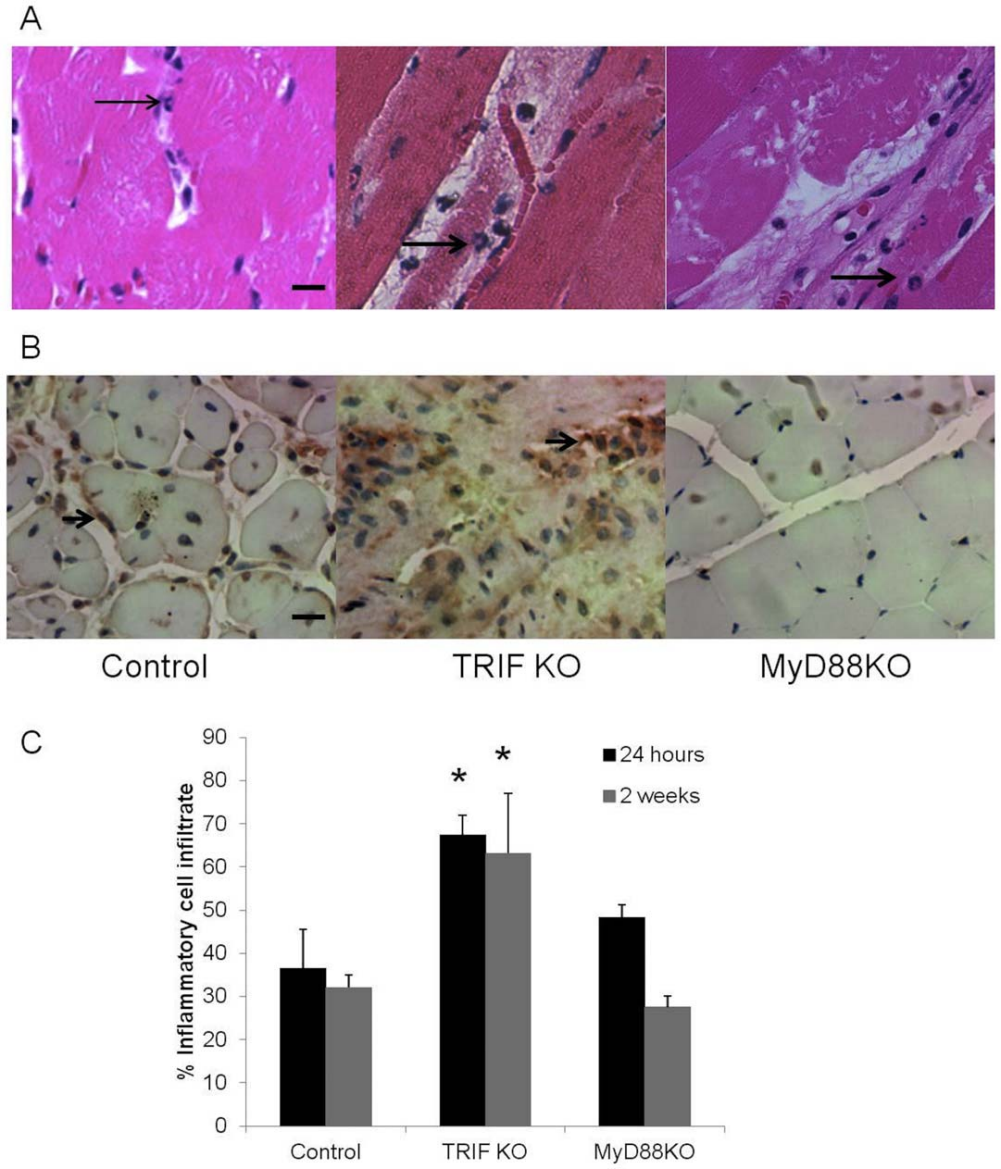
Inflammatory infiltrate is higher in TRIF KO than MyD88KO or control mice. A. PMNs, the predominant inflammatory cell seen at early time points after injury, were quantitated as a percent of total nuclei after 24 hours of ischemia and are demonstrated by arrows; Scale bar = 20 µm. B. Macrophages staining positive for F4/80 (arrow) were quantitated as a percent of total nuclei at two weeks and are demonstrated by arrows; Magnification of 40x. C. Percent of inflammatory cell infiltrate (including PMNs at 24 hours and macrophages at 2 weeks) are demonstrated for control, TRIF KO and Myd88KO mice. *P<0.05, N = 4 each/time point.

**Table 2:** Necrosis, fat replacement and muscle regeneration in control, TRIF KO, and MyD88 KO mice two weeks following femoral artery ligation.

	Necrosis (%)	Fat (%)	Regenerating muscle (%)	Normal Muscle (%)
Control (N = 8)	0	4.8±1.0	95.1±1.0*	0
TRIFKO (N = 4)	41.9±20.0*	0.3±0.2	54.5±21.2	0
MyD88KO (N = 7)	0	0.9±0.6	14.2±13.6	74.9±12.5*

Quantification of area was performed on 4–5 non-overlapping images per section with 3 sections per animal. Results are presented as % of total muscle area (mean ± SEM; *P<0.01).

**Figure 3:**
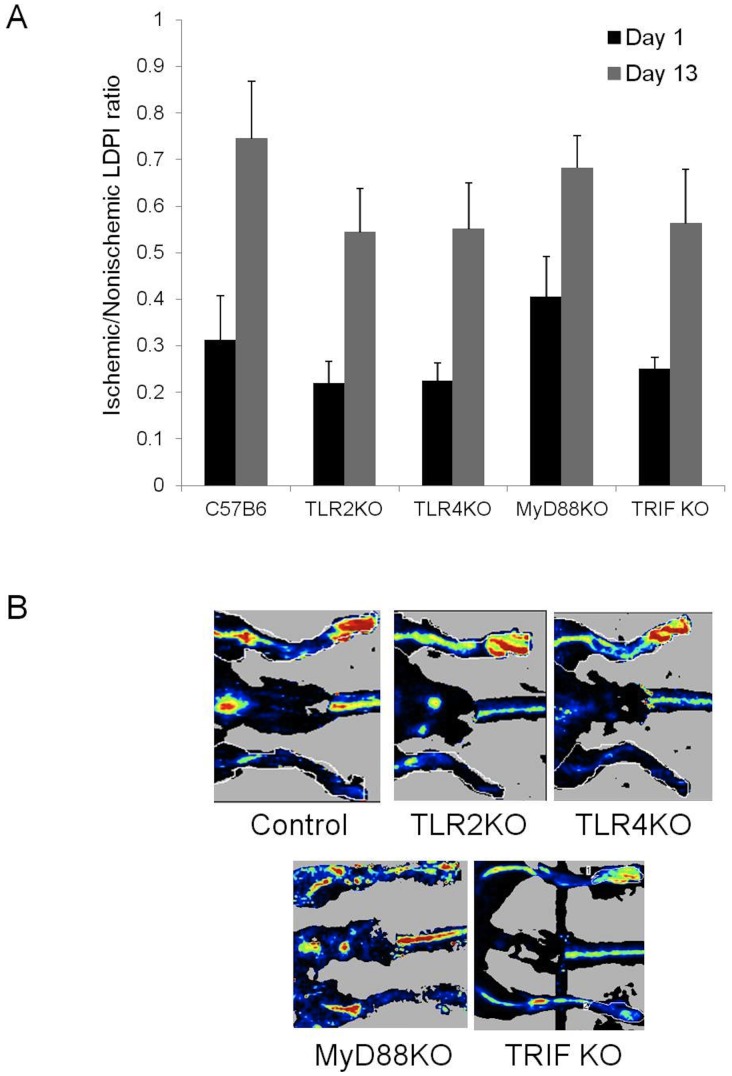
Hind limb perfusion was determined by LDPI measurements and presented as ratios that compared the perfusion of the ischemic to nonischemic limb. (A) The perfusion for control, TLR2 KO, TLR4 KO, MyD88KO and TRIFKO mice at 1 and 13 days after femoral artery ligation. (N = 4–8 per group) is shown (N = 5–7 per group). (B) Representative 13 day LDPI in experimental groups demonstrating extent of perfusion recovery after ischemia.

**Figure 4:**
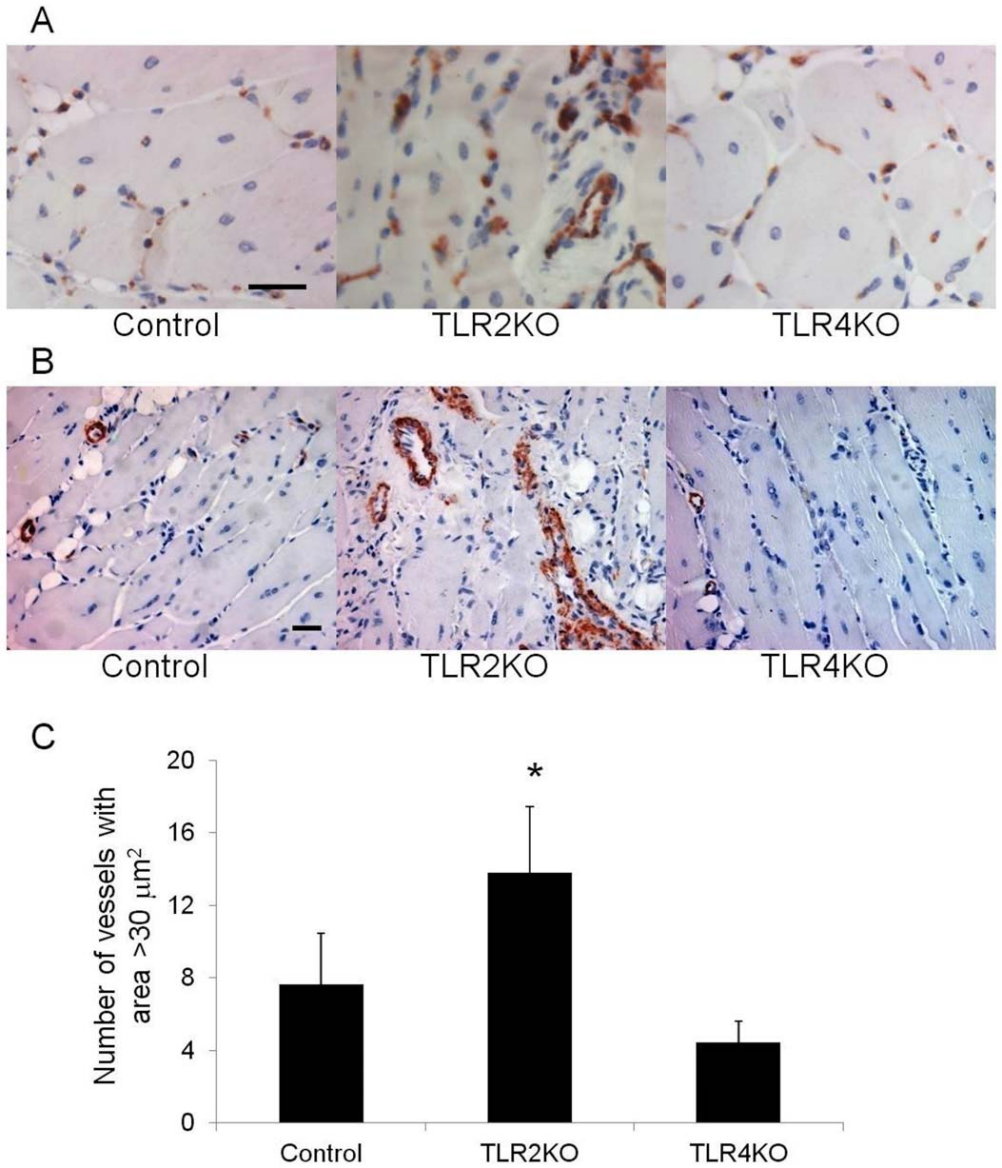
Vascular density in TLR2KO and TLR4KO mice. Representative photomicrographs of CD31 (A) and SMA (B) staining in tibialis anterior muscle from the ischemic hind limb of control, TLR2 KO and TLR4 KO mice two weeks after femoral artery ligation (Scale bar = 25 µm). (C) Quantification of vessels measuring greater than 30 µm^2^ is shown (N = 4 per group; *P<0.05, TLR2 KO vs. TLR4 KO or control).

**Figure 5:**
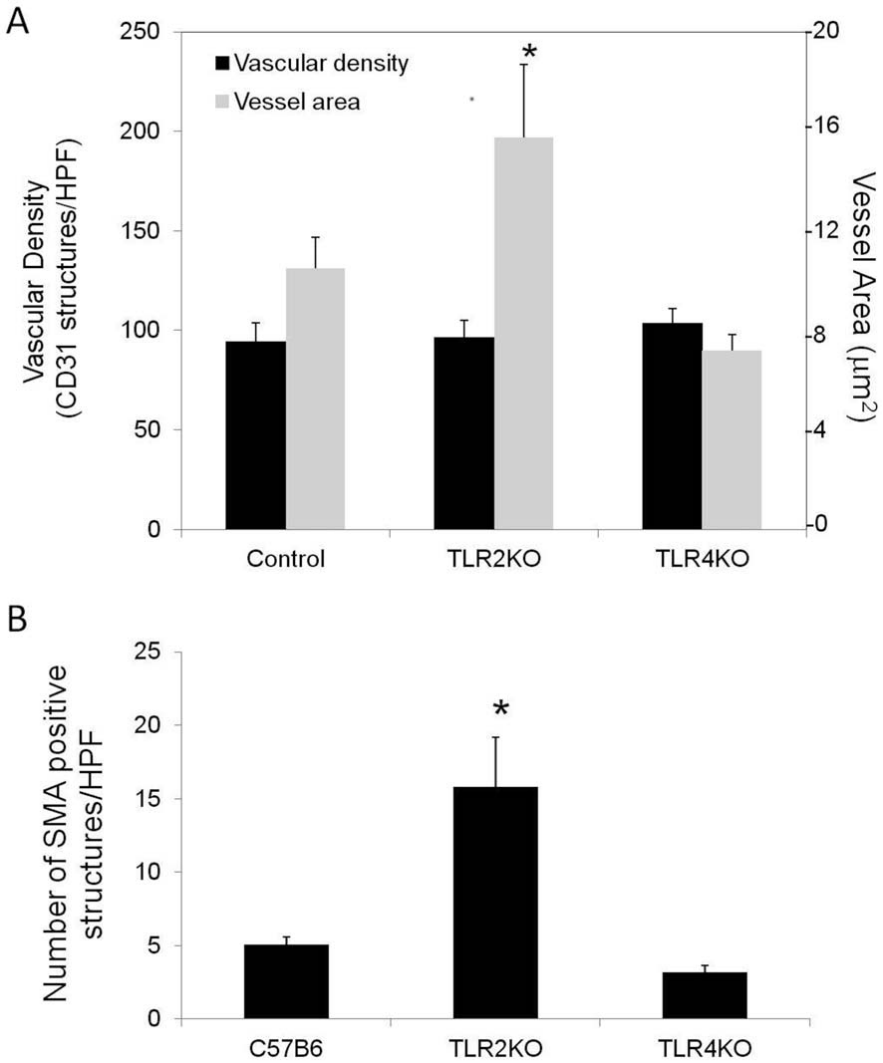
Assessment of the formation of vascular structures following hind limb ischemia. (A) Vascular density was quantified by the number of CD31 structures per HPF in control, TLR2 KO and TLR4 KO mice two weeks following femoral artery ligation (black bars). Vessel area was also measured (gray bar) (N = 4 per group; *P<0.05 versus control and TLR4 KO). (B) Number of SMA staining vessels per HPF 2 weeks (N = 4 per group; *P = 0.003 vs. control and TLR4 KO).

**Figure 6:**
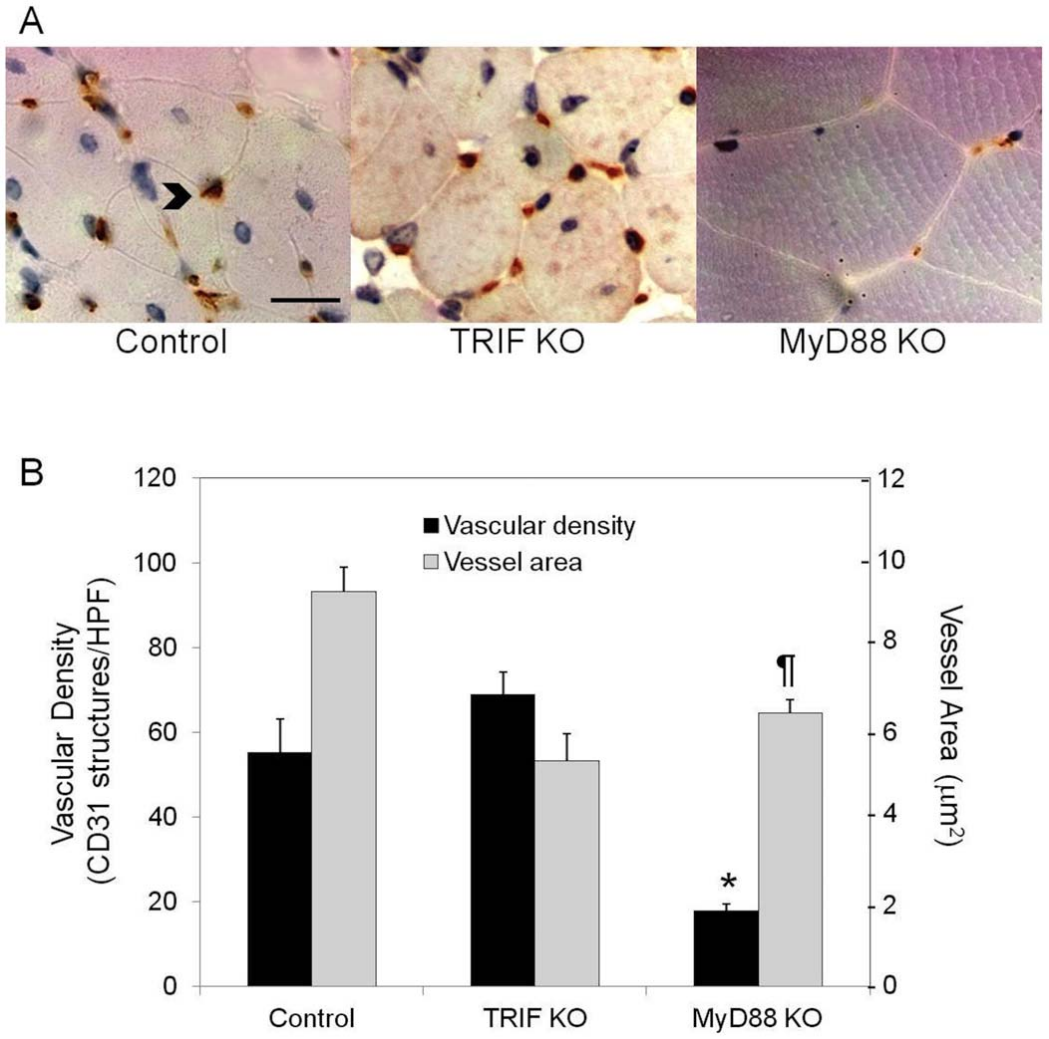
Assessment of vascular structures in control, TRIF KO and MyD88 KO mice. (A) Representative photomicrographs demonstrating CD31 positive structures (arrowhead; scale bar = 25 µm). (B) Quantification of vascular density (black bars) and vessel area (gray bars) are shown (N = 4–8 per group; *P<0.001 vs. TRIF KO and control; ^¶^P = 0.002 vs. control).

**Figure 7:**
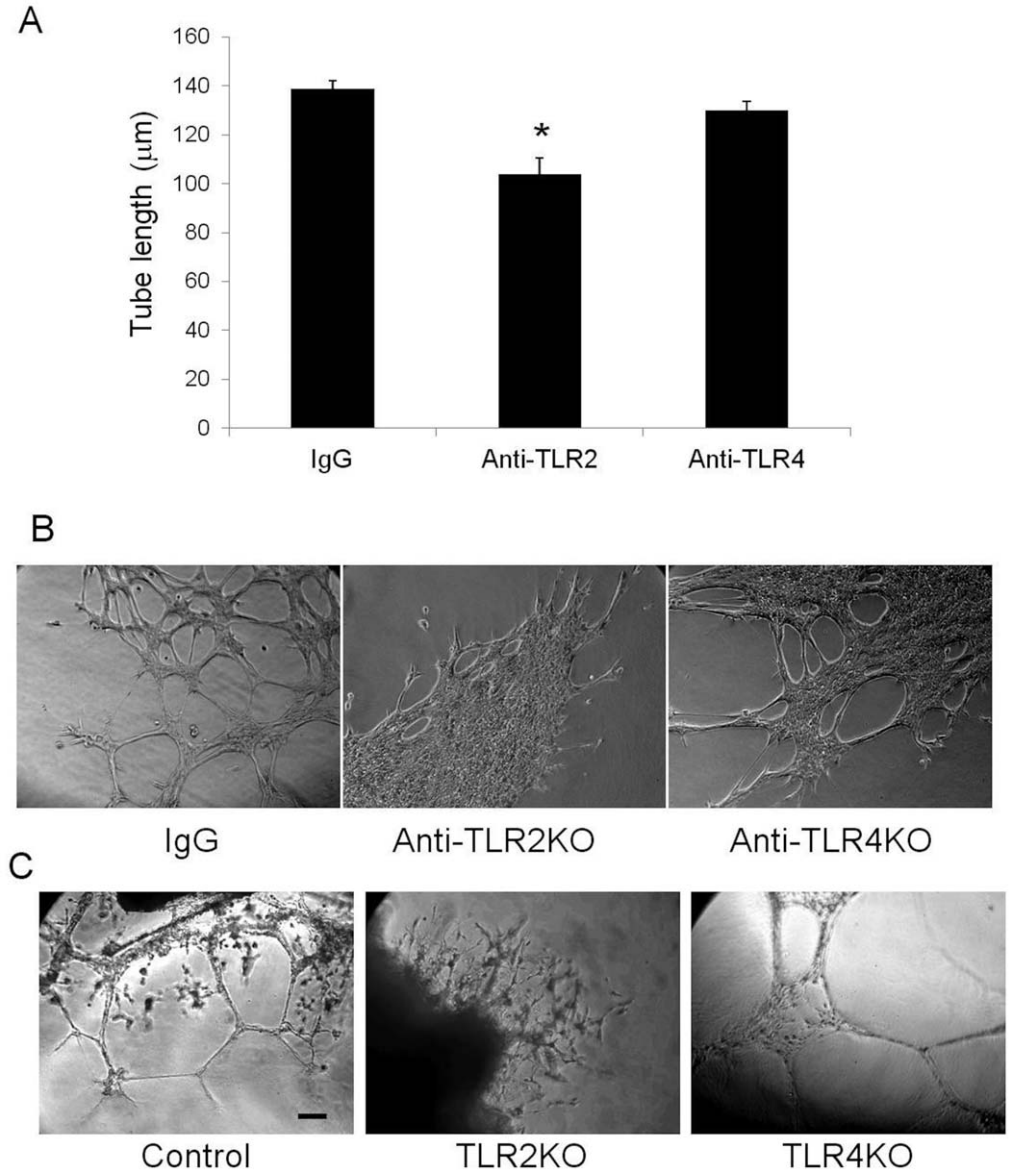
The role of TLR2 and TLR4 in angiogenesis. (A–B) In vitro endothelial tube formation was assessed in HDMVECS seeded on Matrigel treated with nonspecific IgG, anti-TLR2 antibody (T2.5), or anti-TLR4 antibody (HTA 125). Tube formation was quantified after 6 hours by measuring individual tube lengths (4–5 images per well, N = 3 per treatment; *P<0.03 vs. IgG and HTA 125 treated cells). Explants of anterior tibialis muscle from control (N = 5), TLR2 KO and TLR4 KO mice (N = 3) were embedded in Matrigel and cultured in a standard incubator. Endothelial tube sprouting from the muscle explants was imaged at 10x (C) after three weeks of culture (scale bar = 100 µm).

**Figure 8:**
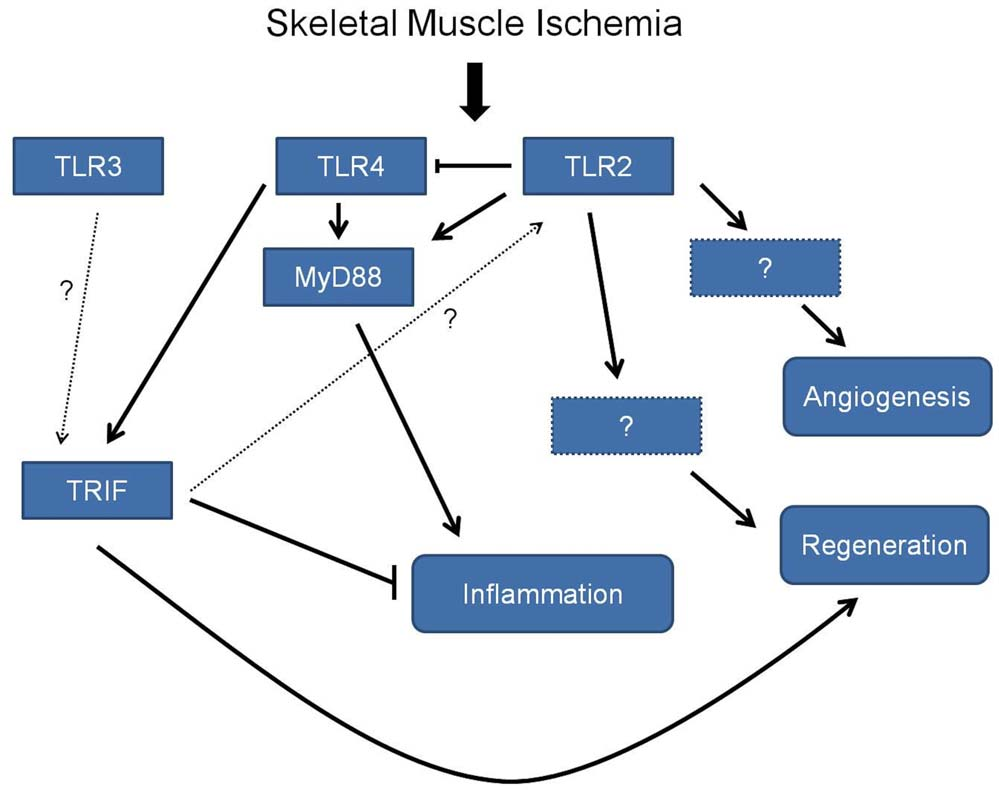
Interplay between TLR4, TLR2, MyD88 and TRIF in mediating inflammation, muscle regeneration and angiogenesis after muscle ischemia.

## Methods

### Reagents

Antibodies for immunohistochemistry and western blot were obtained from the following companies: Santa Cruz, CA (CD31); Jackson Immunoresearch, West Grove, PA (goat-anti-rat secondary antibody), Vector Laboratories, Burlingame, CA (goat-anti-rabbit secondary antibody ABC Elite peroxidase detection system was obtained from Vector Laboratories (Burlingame, CA). AEC chromogen substrate was obtained from ScyTek (Logan,UT). Pierce BCA protein assay kit was obtained from ThermoScientific (Rockford, IL). Human Dermal Microvascular Endothelial Cells (HDMVECs) were obtained from VEC Technologies (Renssalear, NY). Neutralizing antibodies against TLR2 (clone T2.5) and TLR4 (HTA 125) were obtained from Biolegend (San Diego, CA) [18]. The erythrocyte marker TER119 was obtained from Abcam (Cambridge, MA), and isolectin from Sigma (St. Louis,MO). Growth-factor reduced (GFR) and supplemented Matrigel (BD Biosciences, San Jose, CA) was stored at 4°C and allowed to solidify for 30 min in a 37°C standard incubator before use.

### Cell Culture

Human dermal microvascular endothelial cells (HDMVECs) were cultured in a 1∶1 mix of MCDB131 complete media (VEC Technologies) and DMEM with 5% fetal bovine serum (FBS), penicillin, streptomycin (P/S) and L-glutamine. Cells were used between passages 3–12. Growth arrest was performed in DMEM supplemented with 1% FBS.

### Tube Formation

HDMVECs were seeded on GFR Matrigel in 24 well plates in DMEM/1% FBS. After 30 minutes, cells were treated with buffer, nonspecific IgG, neutralizing TLR2 antibody or TLR4 antibody (each 2–µg/1.5 ml) and incubated in normoxic conditions. After 6 hours, cells were fixed in 2% paraformaldehyde, and tube formation was digitally captured with Camera Control Pro 2 software on a Nikon TS100 microscope (Nikon, Melville, NY). Average tube length per well was quantitated using Metamorph software (Downing, PA).

### Muscle Explants

Using a modification of an ex vivo model of angiogenesis, [19] anterior tibialis muscle segments were obtained from TLR2 KO, TLR4 KO and control C57B6 mice (N = 3 each) and embedded into growth factor supplemented Matrigel. Explants were maintained in MCDB-131 basal media plus10%FBS, 150 µg/mL ECGS, 1 µg/mL hydrochlorothiazide, and 10 U/mL heparin. After three weeks, images were captured using a Nikon TS100 microscope with a 4 and 10x objective.

### Animals

Male TLR2 KO, TLR4 KO, MyD88 KO mice, and TRIF KO were used at the 10–12 wks of age and weighed 20–40 gm. The TLR2 KO, MyD88 KO, and TRIF KO mice were generous gifts from Jay Kolls, MD (Children’s Hospital of Pittsburgh, Pittsburgh, PA), Ruslan Medzhitov, PhD (Yale University, New Haven, CT), and Bruce Beutler, (Scripps Research Institute, La Jolla, CA), respectively. The TLR4 KO mice were obtained as a gift from Timothy Billiar, MD (University of Pittsburgh, Pittsburgh, PA). Animals were backcrossed 6–8 times on C57B6 background, and litter mates were used as controls when possible. All procedures conformed to the Guide for the Care and Use of Laboratory Animals published by the United States National Institutes of Health, and were in accordance with the policies of the Institutional Animal Use and Care Committee of the University of Pittsburgh (Approved - protocol #0911093B-5).

### Hind-limb Ischemia Model

Mice were anesthetized with pentobarbitol (0.1 cc/gm IP). Bilateral groins were shaved and prepped with iodine solution. Transverse incisions were made in each groin and the femoral structures, were identified. On the right, the external iliac and femoral veins and arteries and all visible branches were ligated with 6-0 silk, [3] avoiding the femoral nerve. On the left, the femoral vessels were exposed but not ligated. Laser Doppler Perfusion Imaging (LDPI) was performed 1 and 13 days after the initiation of ischemia while mice were anesthetized with inhaled isoflurane. Heart rate and respiratory rate were visually monitored to assess depth of anesthesia, and animals were kept warm with a heating lamp. Excess hairs were removed from the limb with a depilatory cream. The blood flow to both hind-limbs was measured using a Laser Doppler blood-flow meter (PERIMED III, Stockholm, Sweden). Three sequential images were obtained, and averaged. The entire hind-limb starting from the incision site, proximally, to the paw, distally, was incorporated into the region of interest. Perfusion was expressed as a ratio of the ischemic to the non-ischemic leg. In some experiments involving TLR2KO and control C57B6 mice, anterior tibialis muscle was removed at the time of sacrifice, weighed, dried and weighed again to obtain a dry/wet weight ratio as a measurement of vascular permeability. Mice were euthanized by overdose of inhaled isoflurane and cervical dislocation 14 days after ischemic injury.

### Histological Analysis

Tibialis anterior muscle was collected at sacrifice, fixed in formalin, paraffin-embedded and sectioned (8 µm). Sections were stained with hematoxylin and eosin (H&E) for morphologic evaluation. Three H&E sections 60 µm apart were digitally captured using a 20x objective, and evaluated for percent muscle necrosis, muscle regeneration, fat replacement and PMN infiltration using Metamorph (Molecular Devices, Downington, PA), and Image J analysis. PMNs were identified on H and E sections by polymorphonuclear shape as well as eosinophilia. Muscle necrosis was identified by loss of muscle architecture as well as inflammatory infiltrate. Regenerating muscle was characterized by rounded shape and central nuclei [20]. Paraffin-embedded sections were then deparaffinized and immunostained with the erythrocyte marker Ter119 as well as isolectin to identify endothelial cells. Cy-3 conjugated secondary antibody to Ter119 was used for antigen detection to evaluate hemorrhage outside of the vasculature. Corresponding sections were also immunostained for CD31 and SMA to quantify vascular density and vessel morphology, as well as for F4/80 to image macrophage infiltration. After washing, sections were incubated with biotinylated goat-anti-rat secondary antibody. ABC horseradish peroxidase reagent was added after washing. Antigen detection was performed by adding AEC chromogen substrate, and sections were counterstained with hematoxylin. Sections were photographed using a 40X objective. Digital images of each section were color separated using Metamorph, and applied an inclusive threshold to highlight CD31 positive structures. CD31 positive structures per high powered field as well as the area of each vessel were quantified in a blinded and standardized manner for each section using Image J analysis program. Conduit vessels were defined as vessels measuring >30 µm^2^ as described. [21] Inflammatory cell infiltrate was quantified as a percent of total nuclei per 20x image.

### Statistical Analysis

Analysis of variance was used to compare multiple means, whereas T-test was used to evaluate differences between two means. P values <0.05 were considered statistically significant.

## Results

### TLR2, but not TLR4, is Protective after Ischemia in Skeletal Muscle

In response to femoral artery ligation, C57B6 control mice tolerated hind limb ischemia well. The ischemic hind limb exhibited robust muscle regeneration, little fat replacement in the muscle bed, and very good functional recovery at 2 weeks after ischemia. In contrast, TLR2 KO mice developed large areas of muscle necrosis with significantly fewer regenerating myocytes (Figure 1A). Another prominent finding in TLR2 KO mice was multiple areas of hemorrhage within the necrotic muscle (Figure 1B) which was confirmed by dual fluorescent staining. Erythrocytes (orange) were located diffusely within the muscle in TLR2KO mice as opposed to remaining intravascular in TLR4KO and control mouse samples (Figure 1C). In contrast to TLR2KO mice, TLR4 KO mice responded to hind limb ischemia in a fashion very similar to the wild type mice with prominent muscle regeneration, small patches of fat replacement, and little evidence of necrosis (Table 1).

### TRIF is Protective in Hind Limb Ischemia while MyD88 may Mediate Muscle Injury

TLR4 KO mice responded to ischemia with robust muscle regeneration whereas TLR2 KO mice showed marked muscle necrosis. To examine the downstream signaling involved in these divergent responses, we performed femoral artery ligation in MyD88 KO and TRIF KO mice. MyD88 is a signaling pathway utilized by both TLR2 and TLR4 while TRIF is a specific signaling pathway for TLR4 and endolysosomal TLR3. [22] Hind limb ischemia in MyD88 KO mice produced almost no evidence of ischemic or inflammatory damage with very little muscle necrosis or regeneration detected (Figure 1A). Only small islands of fat replacement were identified in a background of normal appearing, mature myocytes. In sharp contrast, TRIF KO mice developed extensive muscle necrosis following femoral artery ligation (Figure 1A). Given that both the response to ischemia as well as the regenerative response likely depends on the degree of inflammation incurred by the injury, inflammatory cell infiltrate was evaluated 1 day and 2 weeks after injury. The percent PMN infiltration was quantified 1 day after injury based on H&E, while F4/80 staining for macrophages was performed at the later time point. PMNs are the predominant inflammatory cell seen at early time points after injury and were easily identifiable by their characteristic nuclear morphology, At both early and late time points, inflammatory cell infiltration was significantly higher in TRIF KO mice compared to control and MyD88 mice. (Figure 2) Of note, while the percent inflammatory cell infiltrate appeared similar at early and later time points, the identity of the cells were different. PMN infiltration predominated 24 hours after ischemia, while macrophages were prominent after two weeks. Additional histological features these mice are summarized in Table 2. The necrotic changes in TRIF KO mice were similar to that observed in the TLR2 KO mice but were much more severe.

### Angiogenesis and Vessel Maturation is Impaired in the Absence of TLR2

By LDPI, there were no differences in perfusion ratios comparing ischemic and nonischemic limbs in all the animal groups studies. This finding was true at both one and 13 days. (Figure 3) Thus, the extensive necrosis in the TLR2 KO and TRIF KO mice did not appear to be due to gross differences in perfusion. When examining the number of CD31 positive structures, there was no difference in the number of vascular structures between wild type, TLR2 KO or TLR4 KO mice in the ischemic limb (Figure 4). However, there were marked morphologic differences in these structures between the strains. In contrast to the other animals studied, TLR2KO mice exhibited a predominance of dilated vessels that stained positive for SMA and were much more consistent with arterioles (Figures 4 and 5). When quantified, TLR2 KO mice had significantly more vessels measuring greater than 30 µm^2^ (Figure 4C) and larger overall vessel area (Figure 5A) than TLR4 KO mice. Additionally, TLR2 KO had significantly more SMA positive vessels than either TLR4 KO or wild type mice (Figure 5B). Based on size measurements, CD31 positive vascular structures in TLR4KO, TRIFKO, MyD88KO and control mice appeared to be consistent with capillaries. Of note, TRIFKO and MyD88KO mice had vessel areas that were significantly smaller than those in controls (Figure 6). MyD88KO mice, which developed limited evidence of ischemic injury, also demonstrated a near normal distribution of vessels with less vascular density than the other strains studied.

Qualitative differences were prominent between the vasculature of TLR2 KO and TLR4 KO mice. This finding, in combination with the observation that tissue hemorrhage was prominent in TLR2KO mice, prompted us to hypothesize that TLR2KO mice had greater vascular fragility or neovessel immaturity. Despite the appearance of hemorrhage within the tissue, wet-dry weights also did not differ among strains (TLR2KO 4.33±0.27 vs. C57B6 4.45±0.09, p = NS) suggesting that vessel permeability was not compromised two weeks after ischemia.

To further examine TLR 2 and 4 function on angiogenic behavior, HDMVECs were cultured on GFR-matrigel in vitro and exposed to antibodies blocking TLR function. Treatment with anti-TLR2 antibody blunted EC tube formation compared to cells treated with control buffer, nonspecific IgG or TLR4 blocking antibody (Figure 7A–B). In a separate angiogenesis assay [23], TLR2 KO tibialis anterior muscle was embedded in GFR-matrigel and allowed to sprout EC tubes. These muscle sections demonstrated poor, blunted EC tube outgrowth after three weeks of culture. In contrast, muscle from wild type mice and TLR4 KO mice developed numerous long tube-like extensions with complex networking (Figure 7C).

## Discussion

Inflammation is critical for angiogenesis in both physiologic processes such as wound healing as well as pathologic processes such as retinopathy and tumor growth. [12], [24], [25] Events that promote inflammation also promote angiogenesis. For example, EC activation with PMA releases P-selectin from Weibel-Palade bodies which then is important in the recruitment of leukocytes. Ang2, stored within EC granules, is also released under the same stimuli [26]. Ang2 is crucial for angiogenesis as it induces vascular permeability, allowing for matrix deposition and endothelial cell migration from existing vessels to form new vascular networks. [12] While it is well known that pattern recognition receptors of the innate immune system activate inflammatory pathways in response to danger signals associated with both tissue injury and pathogen invasion, recent evidence also suggests that TLRs may mediate angiogenic responses associated with wound repair. [27] In ischemia, necrotic cell debris and products of matrix degradation may stimulate innate immune responses predominantly through the TLR2 and TLR4 receptors. Thus, we hypothesized that the muscle recovery and angiogenesis following ischemia are stimulated by similar signals and receptors.

In this study, we evaluated the roles of TLR2 and TLR4 in promoting muscle regeneration and angiogenesis in response to skeletal muscle ischemia. C57B6 mice demonstrated prominent regeneration with little necrosis following ischemia. TLR4 deficiency did not alter this ischemic response pattern with a similar lack of necrosis and abundant regenerating myocytes. In stark contrast, ischemia in the setting of TLR2 deficiency resulted in prominent muscle necrosis with diminished regeneration two weeks after femoral artery ligation. In vitro findings also showed poor endothelial tube sprouting from muscle explants from TLR2 KO mice while TLR4 deficiency did not reduce this angiogenic response. These findings suggest that TLR2 may mediate protective and regenerative processes in skeletal muscle as well as angiogenesis whereas TLR4 signaling does not appear to be essential for these processes. While LDPI did not demonstrate significant differences among the strains, the differences in vessel morphology suggest alterations in the formation of neovessels in response to muscle ischemia that may exist despite a lack of major differences in limb perfusion.

To better understand the involvement of TLR2 and TLR4 in hind limb ischemia, the intracellular signaling pathways for these receptors involving MyD88 and TRIF were examined. MyD88 is a common signaling intermediate for all cell surface TLRs [28], [29] where as TRIF is specific for TLR4 and for endolysosomal TLR3. [22] Interestingly, the absence of MyD88 signaling produced a very protective phenotype with well preserved muscle viability following ischemia that was difficult to discern from the nonischemic hind limb. Inflammatory cell infiltrates were minimal in MyD88 KO mice, and the post-ischemic vascular density was relatively low, suggesting that these animals have either less of an angiogenic drive or an accelerated healing process that returned vascular distribution to normal. These findings contradicted our predictions that MyD88 KO mice would behave similar to TLR2 KO mice and exhibit greater injury. Instead, these data suggest that MyD88 is required for the inflammatory response to ischemia, which may be a critical step in the development of skeletal muscle necrosis and subsequent fat replacement. Alternatively, the findings may suggest an accelerated regeneration process in MyD88KO mice which will be investigated in future studies in our laboratory. Other investigators have reported that inhibition of MyD88 protected against ischemia/reperfusion injury and NFκB translocation in cardiomyocytes. [30] The marked necrosis in TLR2KO mice may ultimately be the result of unopposed TLR4/MyD88 signaling (Figure 8) along with the loss of the regenerative and angiogenic processes mediated by TLR2. MyD88 is also the signaling pathway for other inflammatory mediators such as IL-1 as well as other TLRs. Thus, the significant protective effect of MyD88 deletion strongly supports its predominant function in inflammatory signaling, representing a cumulative effect of multiple innate immune pathways and requires further investigation.

In the absence of TRIF, hind limb ischemia resulted in prominent muscle necrosis similar to observations in the TLR2 KO mice. It has been reported that TLR2 expression can be upregulated in a TLR4-dependent fashion. [31] In our model, a protective role of TLR2 in muscle ischemia may be regulated by TLR4 in a TRIF dependent fashion and can explain why both TLR2 KO and TRIF KO mice developed severe injury in response to ischemia. Our data support a complex role for TLR4 in muscle ischemia; while TLR4 likely mediates marked inflammation through MyD88, it may also mediate protective, regenerative responses by activating TLR2 in a TRIF-dependent fashion. Another mechanism may be that TRIF has a direct effect on muscle regeneration, potentially through a TLR3 or TLR4 mediated pathway. Mathes et al reported that TLR3 mediated cardiac myocyte regeneration following cardiotoxin induced injury. TLR3 is also a mediator in skeletal muscle immunobiology [32], supporting the important role of TLR3 in muscle biology. This complex interplay of different innate immune receptors and signaling pathways as suggested in Figure 8 may represent a system of “checks and balances” that mediates prominent regeneration after ischemia in response to an initial but essential inflammatory response. Future studies will examine the role of TLR3 in the setting of muscle ischemia.

Our data also support an important role for TLR2 in angiogenesis. The small size of the vessels in TLR4KO, MyD88KO and TRIF KO mice suggested the presence of capillaries within the studied tissue. [33] In contrast, TLR2 KO mice developed larger dilated vessels with high SMA content. This finding is more consistent with arteriogenesis, the process by which pre-existing collaterals dilate and mature in response to increased shear stress. [2] It is possible that the absence of a prominent angiogenic response (i.e., capillary formation) in the distal limb of TLR2 KO mice leads to compensatory vessel dilation, immature vascular network formation, impaired gas exchange at the tissue level, and thus muscle necrosis. Alternatively, the prominent muscle injury in TLR2KO mice may be driving an angiogenic response that is ill-equipped to handle the task of supporting regeneration. The significantly impaired endothelial tubing in vitro from explanted TLR2KO skeletal muscle supports this. These explants exhibited markedly blunted tube extensions compared with explants from TLR4 KO or control mice, both of which developed numerous long tubes that established complex networks. A role for TLR2 in angiogenesis has been suggested in a model of oxidative stress. [34].

In summary, our data suggest that mediators of innate immunity, including TLR2 and TLR4, play important roles in muscle regeneration and angiogenesis in the setting of limb ischemia. In particular, there is interplay between TLR2 and TLR4 in determining skeletal muscle survival and regeneration in the setting of ischemia with a different pattern of involvement in the process of angiogenesis. In muscle, TLR2 is associated with protective actions but may depend on TLR4/TRIF signaling. In the vasculature, TLR2 appears to be required for normal angiogenesis. There are independent effects of TLRs on muscle and on vascular structures as evidenced by the striking differences in muscle viability in MyD88 KO and TRIF KO mice which showed similar levels of perfusion recovery. Further studies involving TLR3KO mice, and animals with both endothelial cell specific and inflammatory cell specific knockouts may help elucidate the role of these receptors.
